# Photodynamic Therapy and the Development of Metal-Based Photosensitisers

**DOI:** 10.1155/2008/276109

**Published:** 2008-09-11

**Authors:** Leanne B. Josefsen, Ross W. Boyle

**Affiliations:** Department of Chemistry, The University of Hull, Kingston-upon-Hull HU6 7RX, UK

## Abstract

Photodynamic therapy (PDT) is a treatment modality that has been used in the successful treatment of a number of diseases and disorders, including age-related macular degeneration (AMD), psoriasis, and certain cancers. PDT uses a combination of a selectively localised light-sensitive drug (known as a photosensitiser) and light of an appropriate wavelength. The light-activated form of the drug reacts with molecular oxygen to produce reactive oxygen species (ROS) and radicals; in a biological environment these toxic species can interact with cellular constituents causing biochemical disruption to the cell. If the homeostasis of the cell is altered significantly then the cell enters the process of cell death. The first photosensitiser to gain regulatory approval for clinical PDT was Photofrin. Unfortunately, Photofrin has a number of associated disadvantages, particularly pro-longed patient photosensitivity. To try and overcome these disadvantages second and third generation photosensitisers have been developed and investigated. This Review highlights the key photosensitisers investigated, with particular attention paid to the metallated and non-metallated cyclic tetrapyrrolic derivatives that have been studied *in vitro* and *in vivo*; those which have entered clinical trials; and those that are currently in use in the clinic for PDT.

## 1. PHOTODYNAMIC THERAPY: BACKGROUND

**Figure 1:**
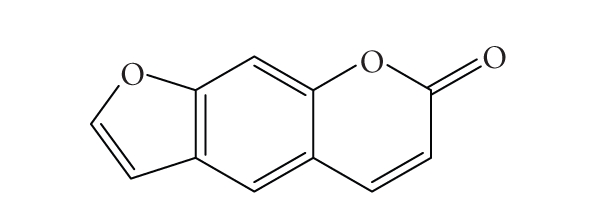
Structure of psoralen.

The use of light in the treatment of disease has been known for many centuries and can be traced back over 4000 years to the ancient Egyptians [1]. The Egyptian people used a combination of the orally ingested Amni Majus plant and sunlight to successfully manage vitiligo: a skin disorder of unknown cause. The active ingredient of this plant (psoralen, Figure 1) is now successfully employed in the worldwide treatment of psoriasis [1–4].

Photodynamic therapy (PDT) is a treatment involving light and a chemical substance (a photosensitiser), used in conjunction with molecular oxygen to elicit cell death. More explicitly, photodynamic therapy is a selective treatment modality for the local destruction of diseased cells and tissue. The selectivity is based on the ability of the photosensitiser to preferentially accumulate in the diseased tissue and efficiently generate singlet oxygen or other highly reactive species such as radicals, which induce target cell death.

**Figure 2:**
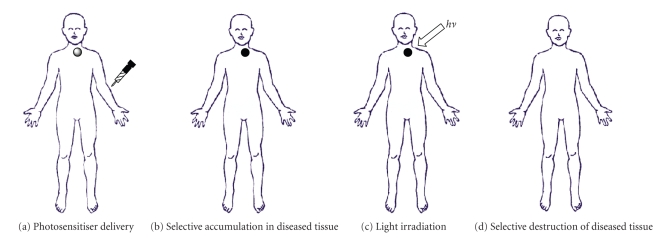
Photosensitiser administration.

The principle of photodynamic therapy is based on a multi-stage process (Figure 2). The first of these stages (Figure 2(a)) sees the administration of a photosensitiser with negligible dark toxicity, either systemically or topically, in the absence of light. When the optimum ratio of photosensitiser in diseased *versus* healthy tissue is achieved, the photosensitiser is (Figure 2(c)) activated by exposure to a carefully regulated dose of light which is shone directly onto the diseased tissue for a specified length of time. The light dose is regulated in order to allow a sufficient amount of energy to be delivered to activate the photosensitiser, but at the same time the dose should be small enough to minimise damage inflicted on neighbouring healthy tissue. It is the activated form of the photosensitiser which evokes a toxic response in the tissue, resulting in cell death. The success of photodynamic therapy lies in the prolonged accumulation of photosensitiser in diseased tissue, relative to the more rapid clearance from normal tissue cells.

Photodynamic therapy is commonly practiced in the treatment of a number of cancers, including those present in the head and neck, the lungs, bladder, and particular skin cancers [5–16]. It has also been successfully used in the treatment of non-cancerous conditions such as age-related macular degeneration (AMD), psoriasis, atherosclerosis, and has shown some efficacy in anti-viral treatments including herpes [2, 3, 5–7, 9, 17–20].

Photodynamic therapy carries advantages for both the patient and the physician: the need for delicate surgery and lengthy recuperation periods is minimised, along with minimal formation of scar tissue and disfigurement. However, photodynamic therapy is not without its drawbacks: a major limitation is the associated general photosensitisation of skin tissue.

## 2. HISTORY OF PHOTODYNAMIC THERAPY

Reports of contemporary photodynamic therapy came first in the investigations led by Finsen in the late nineteenth century [8]. Finsen successfully demonstrated phototherapy by employing heat-filtered light from a carbon-arc lamp (the “Finsen lamp”) in the treatment of a tubercular condition of the skin known as *lupus vulgaris*, for which he won the Nobel Prize in Physiology or Medicine in 1903 [8]. But it was not until the early twentieth century that reports of photodynamic therapy for the treatment of cancer patients (with solid tumours) were made by von Tappeiner's group in Munich [2, 4, 6, 9]. In 1913 another German scientist, Meyer-Betz, described the major stumbling block of photodynamic therapy. After injecting himself with haematoporphyrin (Hp, a photosensitiser), he swiftly experienced a general skin sensitivity upon exposure to sunlight—a problem still persistent with many of todays' photosensitisers [2, 3, 7, 18].

**Figure 3:**
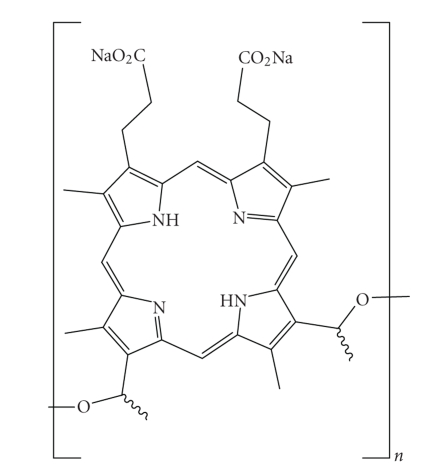
Structure of Photofrin, *n* = 1–9.

Further studies, investigating the accumulation of haematoporphyrin and the purified haematoporphyrin derivative (HpD) in tumours, culminated in the late 1980s with the photosensitiser Photofrin (Figure 3). A photosensitiser which, after further purification, was first given approval in 1993 by the Canadian health agency for use against bladder cancer and later in Japan, USA and parts of Europe for use against certain cancers of the oesophagus and non-small cell lung cancer [3–9, 17, 18, 21, 22].

Photofrin was far from ideal and carried with it the disadvantages of prolonged patient photosensitivity and a weak long-wavelength absorption (630 nm) [6, 7, 21]. This led to the development of improved (second generation) photosensitisers, including Verteporfin (a benzoporphyrin derivative, also known as Visudyne) and more recently, third generation photosensitisers based around targeting strategies, such as antibody-directed photosensitisers [4, 5, 7, 18, 19, 23–25].

## 3. CYCLIC TETRAPYRROLIC CHROMOPHORES AND PHOTOSENSITISERS

**Figure 4:**
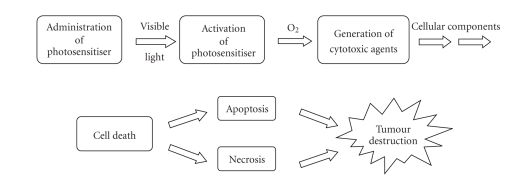
Photosensitiser initiated cell death.

Cyclic tetrapyrrolic molecules are good examples of fluorophores (see Glossary) and photosensitisers. Photosensitisers are molecules, which, when excited by light energy, can utilise the energy to induce photochemical reactions to produce lethal toxic agents. In a cellular environment, these agents (reactive oxygen species (ROS) and radicals) ultimately result in cell death and tissue destruction (Figure 4) [5–9]. Photosensitisers are absorbed into cells all over the body and alone are harmless, that is, in the absence of light, and usually oxygen they have no effect on healthy or abnormal tissue. Ideally, they should be retained by diseased tissue, particularly tumours, for longer periods of time in comparison to healthy tissue; thus it is important to carefully time light exposure and ensure that activation only occurs when the ratio of photosensitiser is greater in diseased tissue than in healthy tissue, thereby minimising unwanted damage to surrounding non-cancerous cells [3, 19].

Photosensitisers also have alternative applications. They have been employed in the sterilisation of blood plasma and water in order to remove blood-borne viruses and microbes and have been considered for agricultural uses, including herbicides and insecticides [5, 9, 26–28].

## 4. PHOTOCHEMISTRY: PHOTOCHEMICAL PROCESSES

Only when a photosensitiser is in its excited state (^3^Psen*) can it interact with molecular oxygen (^3^O_2_) and produce radicals and activated oxygen species (ROS), crucial to the Type II mechanism which is thought to predominate in PDT (see below). These species include singlet oxygen (^1^O_2_), hydroxyl radicals (^•^OH), and superoxide (O_2_^−^) ions and can interact with cellular components including unsaturated lipids; amino acid residues; and nucleic acids. If sufficient oxidative damage ensues, this will result in target-cell death (only within the immediate area of light illumination).

## 5. PHOTOCHEMICAL MECHANISMS

**Figure 5:**
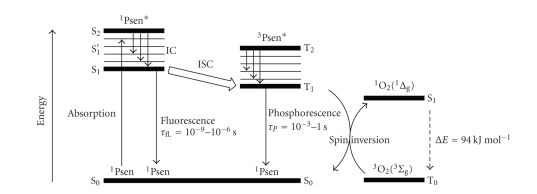
Modified Jablonski energy diagram.

When a chromophore, such as a cyclic tetrapyrrolic molecule, absorbs a photon of electromagnetic radiation in the form of light energy, an electron is promoted into a higher-energy molecular orbital, elevating the chromophore from the ground state (S_0_) into a short-lived, electronically excited state (S_*n*_) composed of a number of vibrational sub-levels (S_*n*_′). The excited chromophore can lose energy by rapidly decaying through these sub-levels *via* internal conversion (IC) to populate the first excited singlet state (S_1_), before quickly relaxing back to the ground state (Figure 5).

The decay from the excited singlet state (S_1_) to the ground state (S_0_) is * via* **fluorescence (S_1_ → S_0_**). Singlet state lifetimes of excited fluorophores are very short (*τ*_fl._ = 10^−9^–10^−6^ seconds) since transitions between the same spin states (S → S or T → T) conserve the spin multiplicity of the electron and, according to the Spin Selection Rules, are therefore considered “allowed” transitions [3, 6, 8]. Alternatively, an excited singlet state electron (S_1_) can undergo spin inversion and populate the lower-energy first excited triplet state (T_1_) *via * intersystem crossing (ISC); a spin-forbidden process, since the spin of the electron is no longer conserved [29–34]. The excited electron can then undergo a second spin-forbidden inversion and depopulate the excited triplet state (T_1_) by decaying back to the ground state (S_0_) *via* **phosphorescence (T_1_ → S_0_)** [29–34]. Owing to the spin-forbidden triplet to singlet transition, the lifetime of phosphorescence (*τ*_*P*_ = 10^−3^ − 1 second) is considerably longer than that of fluorescence.

## 6. PHOTOSENSITISERS AND PHOTOCHEMISTRY

Tetrapyrrolic photosensitisers in the excited singlet state (^1^Psen*, S_>0_) are relatively efficient at undergoing intersystem crossing and can consequently have a high triplet-state quantum yield, Box 2 (Φ_T_ 0.62 (tetraphenylporphyrin (TPP), methanol)), 0.83 (etiopurpurin, benzene), 0.71 (tetrasulphonated TPP, D_2_O), and 0.47 (tetrasulphonated zinc phthalocyanine, methanol)) [8, 35, 36]. The longer lifetime of this species is sufficient to allow the excited triplet state photosensitiser to interact with surrounding bio-molecules, including cell membrane constituents [5, 17].

## 7. PHOTOCHEMICAL REACTIONS

Excited triplet-state photosensitisers can react in two ways defined as Type-I and Type-II processes. Type-I processes can involve the excited singlet or triplet photosensitiser (^1^Psen*, S_1_; ^3^Psen*, T_1_), however due to the short lifetime of the excited singlet state, the photosensitiser can only react if it is intimately associated with a substrate, in both cases the interaction is with readily oxidisable or reducable substrates. Type-II processes involve the direct interaction of the excited triplet photosensitiser (^3^Psen*, T_1_) with molecular oxygen (^3^O_2_, ^3^Σ_g_) [5–8, 17, 18, 37].

**Figure 6:**
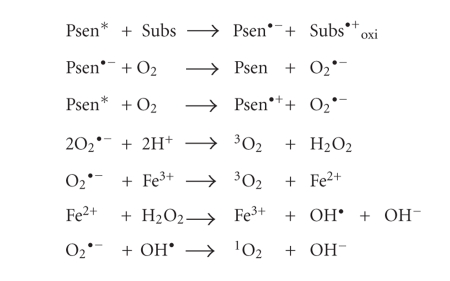
Type-I process (i).

Type-I processes can be divided into two further mechanisms; Type I(i) and Type I(ii). The first of these mechanisms (i) involves the transfer of an electron (oxidation) from a substrate molecule to the excited state photosensitiser (Psen*), generating a photosensitiser radical anion (Psen^•−^) and a substrate radical cation (Subs^•+^). The majority of the radicals produced from Type-I(i) reactions react instantaneously with oxygen, generating a complex mixture of oxygen intermediates. For example, the photosensitiser radical anion can react instantaneously with molecular oxygen (^3^O_2_) to generate a superoxide radical anion (O_2_^•−^), which can go on to produce the highly reactive hydroxyl radical (OH^•^, Figure 6), initiating a cascade of cytotoxic free radicals; this process is common in the oxidative damage of fatty acids and other lipids [17, 18]. Some of the more common Type-I(i) reactions are shown in Figure 6.

**Figure 7:**
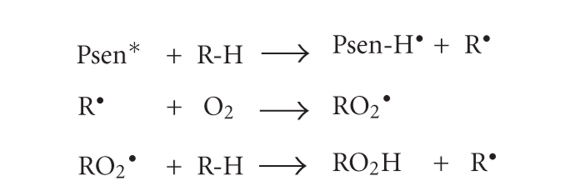
Type-I process (ii).

The second Type-I process (ii) involves the transfer of a hydrogen atom (reduction) to the excited state photosensitiser (Psen*). This generates free radicals capable of rapidly reacting with molecular oxygen and creating a complex mixture of reactive oxygen intermediates, including reactive peroxides (Figure 7). Once again, this can trigger a torrent of cytotoxic events, culminating in cell damage and death.

**Figure 8:**
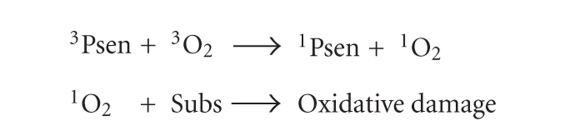
Type-II process.

On the other hand, Type-II processes involve the direct interaction of the excited triplet state photosensitiser (^3^Psen*) with ground state molecular oxygen (^3^O_2_, ^3^Σ_g_, Figure 8); a spin allowed transition—the excited state photosensitiser and ground state molecular oxygen are of the same spin state (T, Figure 5).

**Figure 9:**
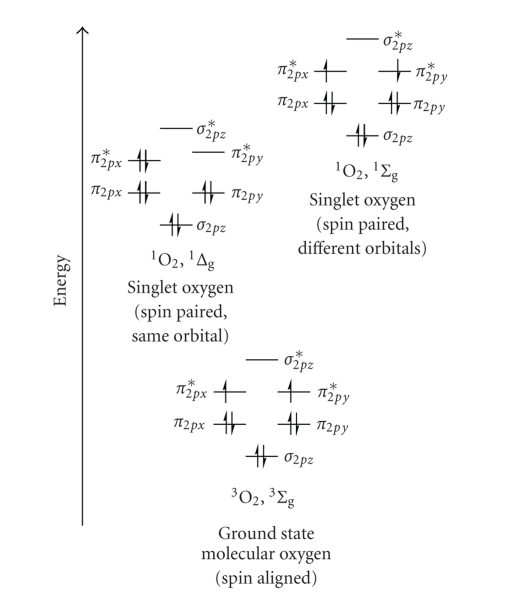
Molecular orbital diagram of oxygen (^3^Σ_g_, ^1^Δ_g_, and ^1^Σ_g_).

When the excited photosensitiser collides with a molecule of molecular oxygen, a process of triplet-triplet annihilation takes place (^3^Psen* → ^1^Psen and ^3^O_2_ *→* ^1^O_2_). This inverts the spin of one of molecular oxygens (^3^O_2_) outermost antibonding electrons, generating two forms of singlet oxygen (^1^Δ_g_ and ^1^Σ_g_, Figure 9), while simultaneously depopulating the photosensitiser's excited triplet state (T_1_ → S_0_, Figure 5). The higher-energy singlet oxygen state (^1^Σ_g_, 157kJ mol^−1^ > ^3^Σ_g_) is very short-lived (^1^Σ_g_ ≤ 0.33 milliseconds (methanol), undetectable in H_2_O/D_2_O) and rapidly relaxes to the lower-energy excited state (^1^Δ_g_, 94kJ mol^−1^ > ^3^Σ_g_) [36]. It is, therefore, this lower-energy form of singlet oxygen (^1^Δ_g_) which is implicated in cell injury and cell death [38].

The highly-reactive oxygen species (^1^O_2_) produced *via* the Type-II process act near to their site of generation and within a radius of action of approximately 20 nm, with a typical lifetime of approximately 40 nanoseconds in biological systems [2, 7, 17]. However, it has recently been suggested that (over a 6 microsecond period) singlet oxygen can diffuse up to approximately 300 nm *in vivo * [39–41]. Singlet oxygen can theoretically only interact with proximal molecules and structures within this radius [17]. ROS are known to initiate a large number of reactions with biomolecules, including amino acid residues in proteins, such as tryptophan; unsaturated lipids like cholesterol and nucleic acid bases, particularly guanosine and guanine derivatives, Box 3, with the latter base more susceptible to ROS [2, 5, 8, 17, 36, 42–45]. These interactions cause damage and potential destruction to cellular membranes and enzyme deactivation, culminating in cell death [8].

It is highly probable that in the presence of molecular oxygen, and as a direct result of the photoirradiation of the photosensitiser molecule, both Type-I and II pathways play a pivotal role in disrupting cellular mechanisms and cellular structure. Nevertheless, there is considerable evidence to suggest that the Type-II photo-oxygenation process predominates in the induction of cell damage, a consequence of the interaction between the irradiated photosensitiser and molecular oxygen [2, 5, 8, 18, 43, 46]. It has been suggested, however, that cells *in vivo * are partially protected against the effects of photodynamic therapy by the presence of singlet oxygen scavengers (such as histidine) and that certain skin cells are somewhat resistant to photodynamic therapy in the absence of molecular oxygen; further supporting the proposal that the Type-II process is at the heart of photoinitiated cell death [17, 43, 47, 48].

**Figure 10:**
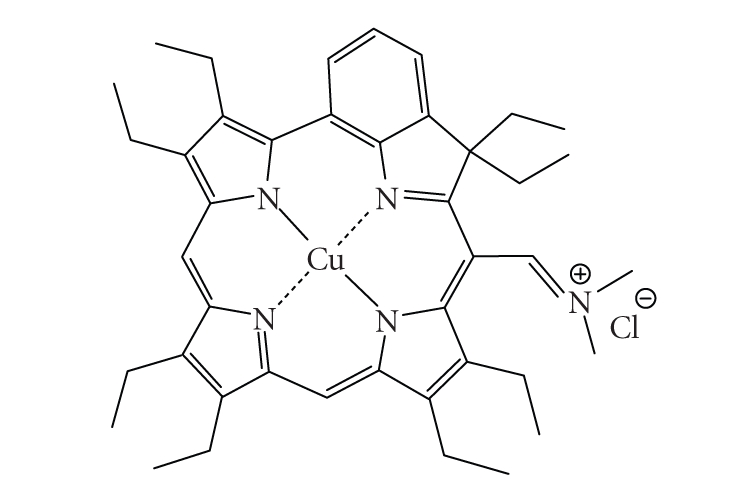
A copper metallated photosensitiser.

The efficiency of Type-II processes is dependent upon the triplet state lifetime *τ*_T_ (see Glossary under luminescence life time) and the triplet quantum yield (Φ_T_) of the photosensitiser. Both of these parameters have been implicated in the effectiveness of a photosensitiser in phototherapeutic medicine; further supporting the distinction between Type-I and Type-II mechanisms. However, it is worthy to note that the success of a photosensitiser is not exclusively dependent upon a Type-II process taking place. There are a number of photosensitisers whose excited triplet lifetimes are too short to permit a Type-II process to occur. For example, the copper metallated octaethylbenzochlorin photosensitiser (Figure 10) has a triplet state lifetime of less than 20 nanoseconds and is still deemed to be an efficient photodynamic agent [13, 43].

## 8. PHOTOSENSITISERS—IDEAL PHOTOSENSITISERS

**Figure 11:**
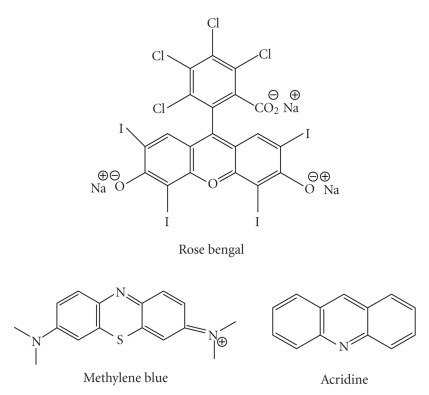
Examples of non-porphyrinic photosensitisers.

**Figure 12:**
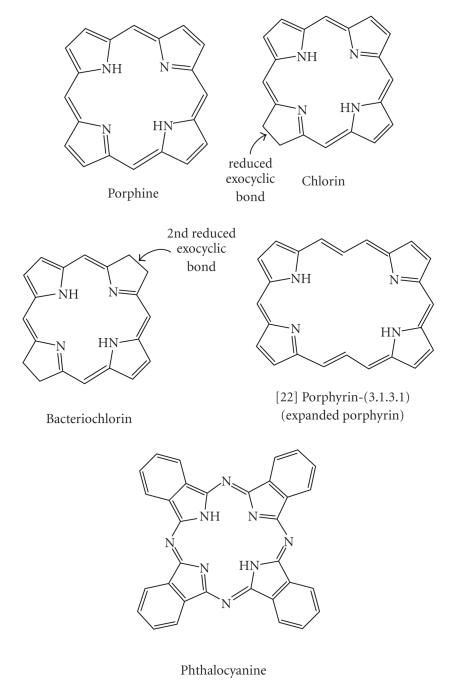
Porphine, chlorine, bacteriochlorin, expanded porphyrin, and phthalocyanine structures.

Although a number of different photosensitising compounds, such as methylene blue (see Glossary), rose bengal, and acridine (Figure 11), are known to be efficient singlet oxygen generators (and therefore potential photodynamic therapy agents), a large number of photosensitisers are cyclic tetrapyrroles or structural derivatives of this chromophore; in particular porphyrin, chlorin, bacteriochlorin, expanded porphyrin, and phthalocyanine (PCs) derivatives (Figure 12). This is possibly because cyclic tetrapyrrolic derivatives have an inherent similarity to the naturally occurring porphyrins present in living matter—consequently they have little or no toxicity in the absence of light [2, 5, 17, 18, 36, 44, 49].

**Figure 13:**
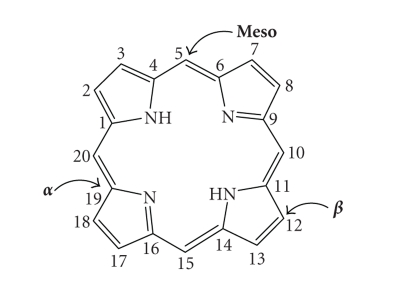
Porphine macrocycle.

Porphyrins are a group of naturally occurring and intensely coloured compounds, whose name is drawn from the Greek word *porphura*, the Greek word for purple [50–52]. These molecules are known to be involved in a number of biologically important roles, including oxygen transport and photosynthesis, and have applications in a number of fields, ranging from fluorescence imaging to medicine [2, 5, 17, 42]. Porphyrins are classified as tetrapyrrolic molecules, with the heart of the skeleton a heterocyclic macrocycle, known as a porphine. The fundamental porphine frame consists of four pyrrolic sub-units linked on opposing sides (*α*-positions, numbered 1, 4, 6, 9, 11, 14, 16, and 19, Figure 13) through four methine (CH) bridges (5, 10, 15, and 20), known as the *meso*-carbon atoms/positions (Figure 13). The resulting conjugated planar macrocycle may be substituted at the *meso*- and/or *β*-positions (2, 3, 7, 8, 12, 13, 17, and 18): if the *meso-* and *β*-hydrogens are substituted with non-hydrogen atoms or groups, the resulting compounds are known as porphyrins.

**Figure 14:**
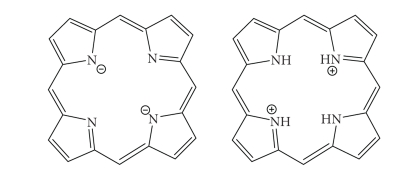
Porphyrin dianionic and dicationic species.

The inner two protons of a free-base porphyrin can be removed by strong bases such as alkoxides, forming a dianionic molecule; conversely, the inner two pyrrolenine nitrogens can be protonated with acids such as trifluoroacetic acid affording a dicationic intermediate (Figure 14). The tetradentate anionic species can readily form complexes with most metals.

## 9. PORPHYRIN ABSORPTION SPECTROSCOPY

**Figure 15:**
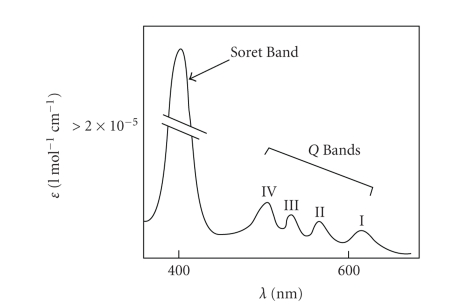
Typical porphyrin absorption spectrum [55, (modified)].

On account of their highly conjugated skeleton, porphyrins have a characteristic ultra-violet visible (UV-VIS) spectrum (Figure 15). The spectrum typically consists of an intense, narrow absorption band (*ε* > 200000 l mol^−1^cm^−1^) at around 400 nm, known as the Soret or B band, followed by four longer wavelength (450–700 nm), weaker absorptions (*ε* > 20000 l mol^−1^cm^−1^ (free-base porphyrins)) referred to as the Q bands [6, 17, 50, 53, 54].

**Figure 16:**
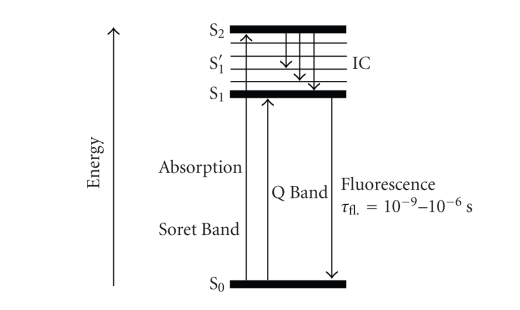
Modified Jablonski energy diagram.

The Soret band arises from a **strong** electronic transition from the (porphyrin) ground state to the second excited singlet state (S_0_ → S_2_, Figure 16); whereas the Q band is a result of a **weak** transition to the first excited singlet state (S_0_ → S_1_). The dissipation of energy *via* internal conversion (IC) is so rapid that fluorescence is only observed from depopulation of the first excited singlet state to the lower-energy ground state (S_1_ → S_0_).

## 10. SECOND-GENERATION PHOTOSENSITISERS

### 10.1. Ideal photosensitiser properties

The key characteristic of any photodynamic sensitiser is its ability to preferentially accumulate in diseased tissue and, *via* the generation of cytotoxic species, induce a desired biological effect. In particular, a good photodynamic sensitiser should adhere to the following criteria:

- have strong absorption with a high extinction coefficient in the red/near infrared region of the electromagnetic spectrum (600–850 nm)—allows deeper tissue penetration [5–7, 17, 36, 43],
- be effective generators of singlet oxygen and other ROS,
- have suitable photophysical characteristics: a high-quantum yield of triplet formation (Φ_T_ ≥ 0.5); a high singlet oxygen quantum yield (Φ_Δ_ ≥ 0.5); a relatively long triplet state lifetime (*τ*_T_, microsecond range); and a high triplet-state energy (≥ 94 KJ mol^−1^) [3, 8, 18, 36, 56]. To date the parameters Φ_T_ = 0.83 and Φ_Δ_ = 0.65 (haematoporphyrin); Φ_T_ = 0.83 and Φ_Δ_ = 0.72 (etiopurpurin); and Φ_T_ = 0.96 and Φ_Δ_ = 0.82 (tin etiopurpurin) have been achieved [2, 36],
- have minimum dark toxicity and negligible cytotoxicity in the absence of light,
- exhibit greater retention in diseased/target tissue over healthy tissue,
- present rapid clearance from the body,
- be single, well-characterised compounds, with a known and constant composition,
- have a short and high yielding synthetic route (with easy translation into multi-gram scales/reactions),
- have a simple and stable drug formulation,
- be soluble in biological media, allowing direct intravenous administration and transport to the intended target. Failing this, a hydrophilic delivery system should be sought enabling efficient and effective transportation of the photosensitiser to the target site *via* the bloodstream.

While the major disadvantages associated with the first generation photosensitisers HpD and Photofrin (skin sensitivity and weak absorption at 630 nm) have not prevented the treatment of some cancers and other diseases, they have markedly reduced the successful application of these photosensitisers to a wider field of disease. The development of second generation photosensitisers, designed to minimise the drawbacks of the first generation photosensitisers, was key to the development of photodynamic therapy. A number of new photosensitisers were therefore developed to overcome these short comings.

**5-Aminolaevulinic acid**

**Scheme 1:**
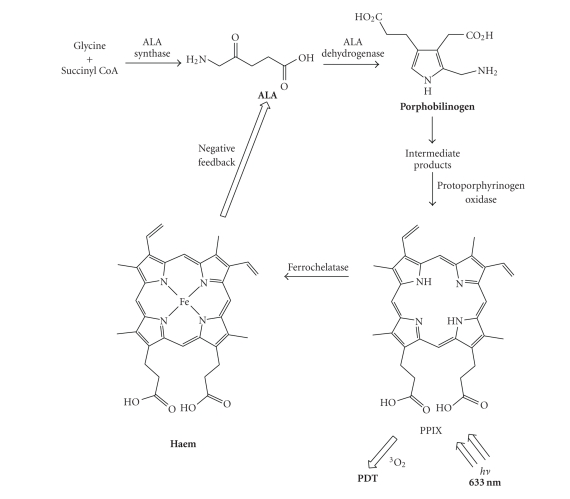
Simplified haem biosynthesis.

The 5-Aminolaevulinic acid (ALA) is a prodrug used in the clinic to treat and image a number of superficial cancers and tumours (see Tables 2 and 3) [5–9, 11, 17, 18]. ALA on its own is not a photosensitiser, but a key precursor in the biosynthesis of the naturally occurring porphyrin, haem (Scheme 1).

Haem is synthesised in every energy-producing cell in the body and is a key structural component of haemoglobin, myoglobin, and other haemproteins. The immediate precursor to haem is protoporphyrin IX (PPIX), an effective photosensitiser. Haem itself is not a photosensitiser, due to the coordination of a paramagnetic ion (iron; see Glossary; see also diamagnetic species) in the centre of the macrocycle, causing significant reduction in excited state lifetimes [5–9, 11].

The haem molecule is synthesised from glycine and succinyl coenzyme A (succinyl CoA). The rate-limiting step in the biosynthesis pathway is controlled by a tight (negative) feedback mechanism in which the concentration of haem regulates the production of ALA. However, this controlled feedback can be by-passed by artificially adding excess exogenous ALA to cells. The cells respond by producing PPIX (photosensitiser) at a faster rate than the ferrochelatase enzyme can convert it to haem [5–9, 11, 17, 18].

ALA, marketed as Levulan (DUSA Pharmaceuticals Incorporated, Toronto, Canada), has shown promise in photodynamic therapy (tumours) *via * both intravenous and oral administration, as well as through topical administration in the treatment of malignant and non-malignant dermatological conditions, including psoriasis, Bowen's disease, and Hirsutism (Phase II/III clinical trials, see Glossary) [5–9, 11, 18].

ALA shows a more rapid accumulation in comparison to other intravenously administered sensitisers [5–9, 11]. Typical peak tumour accumulation levels post-administration for PPIX are usually achieved within several hours; compare this with other (intravenously administered) photosensitisers which may take up to 96 hours to reach peak levels and one of the main advantages of ALA can be clearly seen. ALA is also excreted more rapidly from the body (∼24 hours) than other photosensitisers, minimising patient photosensitivity [5–8, 11].

In an attempt to overcome the poor bioavailability when ALA is applied topically, esterified ALA derivatives with improved pharmacological properties have been examined [5–8, 11]. A methyl ALA ester (Metvix) is now being marketed by Photocure ASA (Oslo, Norway) as a potential photosensitiser for basal cell carcinoma and other skin lesions [5, 6, 9, 17]. Benzyl (Benvix) and hexyl ester (Hexvix) derivatives are also registered by Photocure ASA for the treatment of gastrointestinal cancers and for the diagnosis of bladder cancer [9].

**Verteporfin**

**Figure 17:**
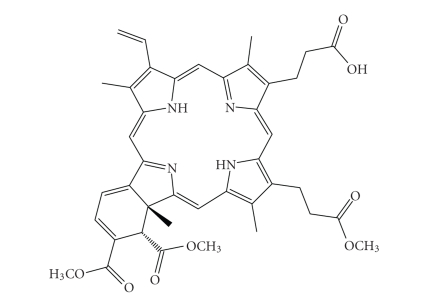
Verteporfin.

The second generation photosensitiser, benzoporphyrin derivative monoacid ring A (BPD-MA, Figure 17) has been developed by QLT Phototherapeutics (Vancouver, Canada) under the trade name Visudyne (Verteporfin, for injection) and, in collaboration with Ciba Vision Corporation (Duluth, GA, USA), has undergone Phase III clinical trials (USA) for the photodynamic treatment of wet age-related macular degeneration (AMD, see Glossary) and cutaneous non-melanoma skin cancer [3, 5–7, 9, 57–59]. Verteporfin is currently marketed by Novartis Pharmaceuticals Corporation (NJ, USA).

The chromophore of BPD-MA has a red-shifted and intensified long-wavelength absorption maxima at approximately 690 nm. Tissue penetration by light at this wavelength is 50% greater than that achieved for Photofrin (*λ*_max._ = 630 nm) [5, 60].

Verteporfin has further advantages over the first generation sensitiser Photofrin. It is rapidly absorbed by the tumour (optimal tumour-normal tissue ratio 30–150 minutes post-intravenous injection) and is rapidly cleared from the body, minimising patient photosensitivity (1-2 days) [5, 61].

**Purlytin**

**Figure 18:**
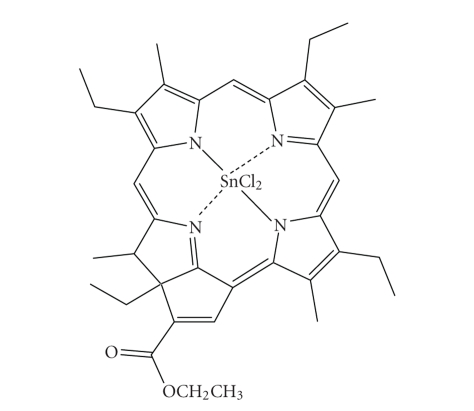
Purlytin.

Tin etiopurpurin, a chlorin photosensitiser (Figure 18), is marketed under the trade name Purlytin by Miravant Medical Technologies (Santa Barbara, Calif, USA) [5–9, 62]. Purlytin has also undergone Phase II clinical trials (USA) for cutaneous metastatic breast cancer and Kaposi's sarcoma in patients with AIDS (acquired immunodeficiency syndrome) [3, 7]. Purlytin has been used successfully to treat the non-malignant conditions psoriasis and restenosis [5].

Chlorins (Figure 12) are distinguished from the parent porphyrins by a reduced exocyclic double bond. The result of the reduced bond is a decrease in the symmetry of the conjugated macrocycle, leading to an increased absorption in the long-wavelength portion of the visible region of the electromagnetic spectrum (650–680 nm). More correctly, Purlytin is a purpurin; a degradation product of chlorophyll [8, 9, 12].

Purlytin has a tin atom chelated in its central cavity which causes a red-shift of approximately 20–30 nm (with respect to Photofrin and non-metallated etiopurpurin, *λ*_max._SnEt_2_ = 650 nm) [6, 9, 12]. Purlytin has been reported to localise in skin and produce a photoreaction 7–14 days post-administration [6, 9].

**Foscan**

**Figure 19:**
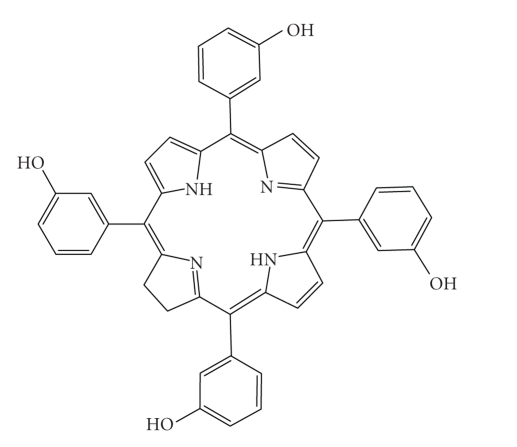
Foscan.

Tetra(*m*-hydroxyphenyl)chlorin (*m*THPC, Figure 19) has been developed and entered into clinical trials (USA and Europe) under the trade name Foscan by Scotia Pharmaceutics (Guildford, Surrey, UK) and BioLitec Pharma Limited (Dublin, Ireland) [3, 5–9, 11, 18]. Foscan, also known as Temoporfin, has been evaluated as a phototherapeutic agent against head and neck cancers in these trials [5]. It has also been investigated in clinical trials for malignant and non-malignant diseases, including gastric and pancreatic cancers, hyperplasia, field sterilisation after cancer surgery and for the control of antibiotic-resistant bacteria, in the USA, Europe, and the Far East [5, 9, 11].

Foscan has a singlet oxygen quantum yield comparable to other chlorin photosensitisers but the low drug and light doses (approximately 0.1 mg kg^−1^ and as low as 5 J cm^−2^, resp.) required to achieve photodynamic responses (equivalent to Photofrin, 2–5 mg kg^−1^, 100–200 J cm^−2^; therefore Foscan is approximately 100 times more photoactive than Photofrin), potentially make Foscan one of the most potent second generation photosensitisers currently under investigation [5, 7, 9].

Unfortunately, Foscan can render patients photosensitive for up to 20 days after initial illumination [6, 63, 64]. One solution to this problem would be to use lower drug doses.

**Lutex**

**Figure 20:**
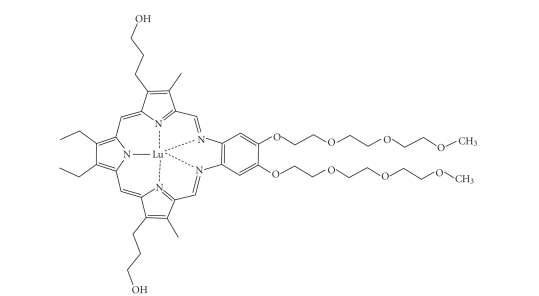
Lutex.

Lutetium texaphyrin, marketed under the trade name Lutex and Lutrin (Pharmacyclics, Calif, USA), is a “texas-sized” porphyrin [5–9, 18, 65, 66]. Texaphyrins (first synthesised in 1987 by Sessler and his group) are expanded porphyrins that have a penta-aza core (Figure 20). The result of this macrocyclic modification is a strong absorption in the 730–770 nm region of the electromagnetic spectrum [9, 12]. This region is particularly important since tissue transparency is optimal in this range. As a result, Lutex-based PDT can (potentially) be carried out more effectively at greater depths and on larger tumours [5, 6].

Lutex has entered Phase II clinical trials (USA) for evaluation against breast cancer and malignant melanomas [6, 67].

A Lutex derivative, Antrin, has also undergone Phase I clinical trials (USA) for the prevention of restenosis (see Glossary) of vessels after cardiac angioplasty by photoinactivating foam cells that accumulate within arteriolar plaques [6, 68]. A second Lutex derivative, Optrin, is in Phase I trials for AMD [5].

Texaphyrins are being developed further by Pharmacyclics as radiosensitisers (Xcytrin, see Glossary) and chemosensitisers (see Glossary) [5]. Xcytrin, a gadolinium texaphyrin (motexafin gadolinium), has been evaluated in Phase III clinical trials against brain metastases and Phase I clinical trials (USA) for primary brain tumours [5].

**ATMPn**

**Figure 21:**
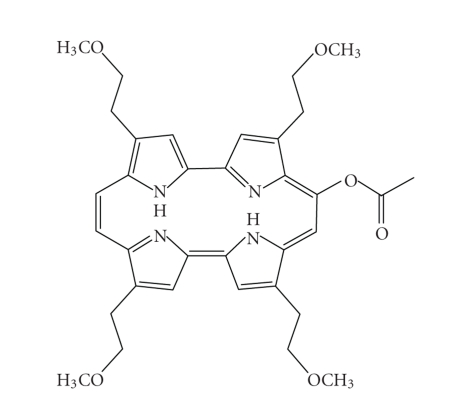
ATMPn.

9-Acetoxy-2,7,12,17-tetrakis-(*β*-methoxyethyl)-porphycene (Figure 21) has been evaluated by Glaxo Dermatology (GlaxoWellcome, NC, USA) and Cytopharm (Calif, USA) as a photodynamic therapy agent for dermatological applications against psoriasis vulgaris and superficial non-melanoma skin cancer [5, 69–72].

**Zinc phthalocyanine CGP55847**

**Figure 22:**
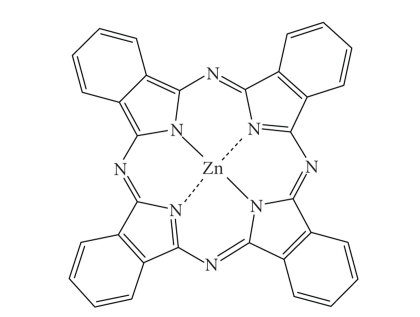
Zinc phthalocyanine CGP55847.

A liposomal formulation of zinc phthalocyanine (CGP55847, Figure 22), developed by QLT Phototherapeutics (Vancouver, Canada) and sponsored by Ciba Geigy (Novartis, Basel, Switzerland), has undergone clinical trials (Phase I/II, Switzerland) against squamous cell carcinomas of the upper aerodigestive tract [5, 18, 73, 74]. Phthalocyanines (PCs) (Figure 12) are related to tetra-aza porphyrins. Instead of four bridging carbon atoms at the *meso-*positions, as for the porphyrins, PCs have four nitrogen atoms linking the pyrrolic sub-units together. PCs further differ from porphyrins through the presence of an extended conjugate pathway: a benzene ring is fused to the *β*-positions of each of the four-pyrrolic sub-units. These benzene rings act to strengthen the absorption of the chromophore at longer wavelengths (with respect to porphyrins). The absorption band of PCs is almost two orders of magnitude stronger than the highest Q band of haematoporphyrin [12]. These favourable characteristics, along with the ability to selectively functionalise their peripheral structure, make PCs favourable photosensitiser candidates [10, 75–78].

**Figure 23:**
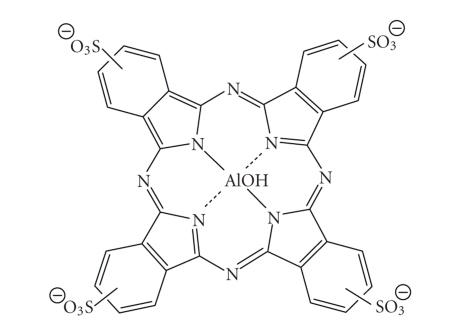
Photosense.

A sulphonated aluminium PC derivative (Photosense, Figure 23) has also entered clinical trials (Russian Academy of Medical Sciences, and the surgical clinic of the Moscow Medical Academy, Moscow, Russia) against skin, breast, and lung malignancies and cancer of the gastrointestinal tract [5, 18, 79–81]. Sulphonation significantly increases PC solubility in polar solvents including water, circumventing the need for alternative delivery vehicles [9, 12, 18, 82].

A third PC under investigation is a silicon complex, PC4. This photosensitiser is being examined for the sterilisation of blood components at the New York Blood Centre (VI Technologies Incorporated (Vitex), Melville, NY, USA), against human colon, breast, and ovarian cancers and against glioma [5, 83–89].

A shortcoming of many of the metallo-PCs is their tendency to aggregate in aqueous buffer (pH 7.4), resulting in a decrease, or total loss, of their photochemical activity. This behaviour can be minimised in the presence of detergents [12].

Metallated cationic porphyrazines (PZ), including PdPZ^+^, CuPZ^+^, CdPZ^+^, MgPZ^+^, AlPZ^+^, and GaPZ^+^, have been developed and also tested *in vitro * on V-79 (Chinese hamster lung fibroblast) cells. Results have suggested these photosensitisers are capable of inducing substantial dark toxicity [12].

**Naphthalocyanines**

**Figure 24:**
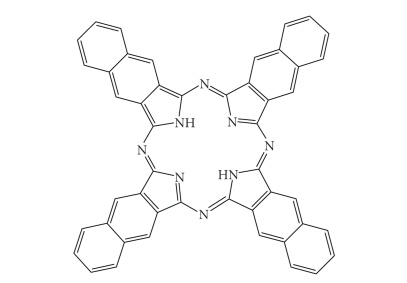
A naphthalocyanine.

Naphthalocyanines (NCs, Figure 24) are an extended PC derivative. They have an additional benzene ring attached to each isoindole sub-unit on the periphery of the PC structure. Subsequently, NCs absorb strongly at even longer wavelengths (approximately 740–780 nm) than PCs (670–780 nm), further increasing the depth NC photosensitisers can be effectively used at. This absorption in the near infrared region makes NCs good candidates for photodynamic treatment of highly pigmented tumours, including melanomas, which present significant problems with respect to transmission of visible light.

However, a number of problems are associated with NC photosensitisers. NCs are generally less stable than their PC relatives: they readily decompose in the presence of light and oxygen; and metallo-NCs, which lack axial ligands, have a tendency to form H-aggregates in solution [12, 90]. These aggregates are photoinactive, thus compromising the photodynamic efficacy of NCs [12]. The main investigations into NCs as photodynamic therapy agents have been carried out by Kenney and co-workers, van Lier's group and the Bulgarian Academy of Sciences (Sofia, Bulgaria) (see below).

**Functional groups**

Altering the peripheral functionality of porphyrin-type chromophores can also have an effect on photodynamic activity.

Diamino platinum porphyrins show high anti-tumour activity, demonstrating the combined effect of the cytotoxicity of the platinum complex and the photodynamic activity of the porphyrin species [12, 91].

Positively charged PC derivatives have also been investigated [12, 64, 76, 77]. Cationic species are believed to selectively localise in the vital sub-cellular organelle, the mitochondrion. Mitochondria are key to the survival of a cell; being the site of oxidative phosphorylation, and hence are potentially important PDT targets.

Zinc and copper cationic derivatives have been investigated. Although, the positively charged zinc complexed PC was found to be less photodynamically active than its neutral counterpart *in vitro* against V-79 cells [12].

**Figure 25:**
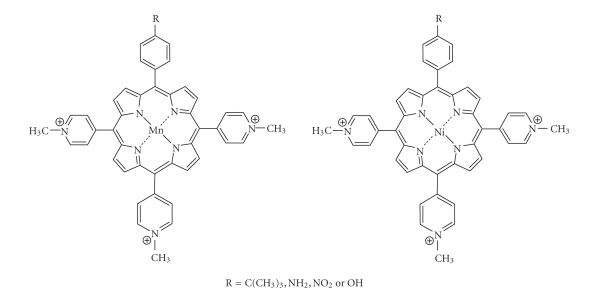
Water-soluble cationic metallated porphyrins.

Water-soluble cationic porphyrins bearing nitrophenyl, aminophenyl, hydroxyphenyl, and/or pyridiniumyl functional groups exhibit varying cytotoxicity to cancer cells *in vitro *, depending on the nature of the metal ion (Mn, Fe, Zn, Ni), and on the number and type of functional groups [12, 77, 92]. The manganese pyridiniumyl derivative has shown the highest photodynamic activity, while the nickel analogue is photoinactive (Figure 25) [12, 92].

Another metallo-porphyrin complex, the iron chelate, was found to be more photoactive (towards HIV and simian immunodeficiency virus in MT-4 cells) than the manganese complexes; the zinc derivative was found to be photoinactive [12, 93].

**Figure 26:**
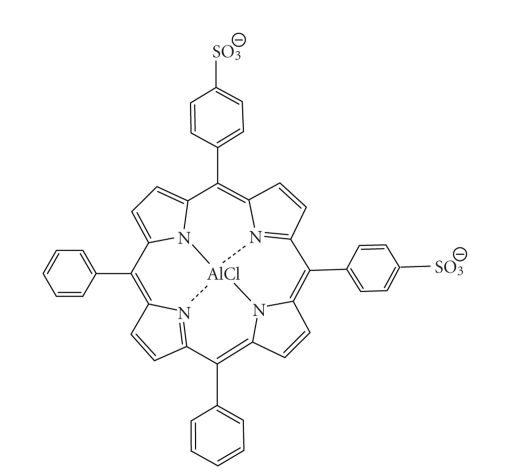
5,10-Di-(4-sulphonatophenyl)-15,20-diphenylporphyrinato aluminium chloride.

The hydrophilic sulphonated porphyrins and PCs (AlPorphyrin and AlPC) compounds were tested for photodynamic activity [94]. The disulphonated analogues (with adjacent substituted sulphonated groups, Figure 26) exhibited greater photodynamic activity than their di-(symmetrical), mono-, tri- and tetra-sulphonated counterparts; tumour activity increased with increasing degree of sulphonation [8, 78].

## 11. THIRD-GENERATION PHOTOSENSITISERS

The poor solubility of many photosensitisers in aqueous media, particularly at physiological pH, prevents their intravenous delivery directly into the bloodstream. It would be advantageous therefore, if a delivery model could be conceived which would allow the transportation of these (otherwise potentially useful) photosensitisers to the site of diseased tissue.

Work has recently focused on designing systems to effect greater selectivity and specificity on the photosensitiser in order to enhance cellular uptake [7, 38]. A number of possible delivery strategies have been suggested, ranging from the use of oil-in-water (o/w) emulsions to liposomes and nanoparticles as potential carrier vehicles [3, 7, 18, 36, 95, 96]. There is concern however, that although the use of these systems may increase the therapeutic effect observed as a result of photodynamic therapy, the carrier system may inadvertently decrease the “observed” singlet oxygen quantum yield (Φ_Δ_) of the encapsulated photosensitiser: the singlet oxygen generated by the photosensitiser would have to diffuse out of the carrier system; and since it (singlet oxygen) is believed to have a narrow radius of action, singlet oxygen may not reach the target and elicit its desired effect [18]. It may also be possible that, if the size of the carrier is not sufficiently small or that the carrier system does not fully dissolve in physiological media, the incidence/exciting light may not be appropriately absorbed and light scattering may be significant, thus inadvertently reducing the singlet oxygen yield. An alternative delivery method which would remove this problem is the use of targeting moieties. Typical targeting strategies have included the investigation of photosensitisers directly attached to biologically active molecules such as antibodies [23–25]. These third generation photosensitisers are currently showing promise *(in vitro)* against colorectal tumour cells [24].

**Metallation**

A wide range of metals have been used to form complexes with photosensitiser macrocycles, with variable photodynamic results. A number of the second generation photosensitisers described earlier contain a chelated central metal ion. The main metals which have been used are transition metals, although a number of photosensitisers co-ordinated to group 13 (Al, AlPcS_4_) and group 14 (Si, SiNC, and Sn, SnEt_2_) metals have also been synthesised.

There seems to be no consistent observation as to the potential success of metallated photosensitisers. Indeed, a wide range of photosensitisers are metallated, but the metal ion does not confer definite photoactivity on the photosensitiser. Copper (II), cobalt (II), iron (II), and zinc (II) complexes of Hp are all photoinactive in contrast to metal-free porphyrins [12]. Yet the reverse has been observed for texaphyrin and PC photosensitisers; only the metallo-complexes have demonstrated efficient photosensitisation [12].

The presence and nature of the central metal ion, bound by a number of photosensitisers, strongly influences the photophysical properties of the photosensitiser [12, 64, 77]. Chelation of paramagnetic metals to a PC chromophore appears to shorten triplet lifetimes (down to nanosecond range), generating variations in the triplet quantum yield and triplet lifetime of the photoexcited triplet state of the metallated PC (mPC) [12, 64, 77, 97].

Intersystem crossing (ISC) is an important parameter of photosensitisers. The triplet quantum yield and lifetime of a photosensitiser are directly related to the efficiency of singlet oxygen generation; a key component in the success of a photosensitiser [97].

Certain heavy metals are known to enhance ISC. Generally, diamagnetic metals promote ISC and have a long triplet lifetime [64, 77, 97]. In contrast, paramagnetic species deactivate excited states, reducing the excited-state lifetime and preventing photochemical reactions from taking place [97]. However, there are well-known exceptions to this generalisation, including copper octaethylbenzochlorin [13].

For many of the metallated paramagnetic texaphyrin species, triplet-state lifetimes are down in the nanosecond range [97]. These results are also mirrored by metallated PCs. PCs metallated with diamagnetic ions, such as Zn^2+^, Al^3+^, and Ga^3+^, generally yield photosensitisers with desirable quantum yields and lifetimes (Φ_T_ 0.56, 0.50 and 0.34 and *τ*_T_ 187, 126 and 35 *μ*s, resp.) [12, 97]. The ZnPC photosensitiser (ZnPcS_4_) has a singlet oxygen quantum yield of 0.70; nearly twice that of most other mPCs (Φ_Δ_ at least 0.40) [12, 18]. Hence, the latter diamagnetic complexes should be strong candidates for PDT.

Since the heavy metal effect (see Glossary) is known to promote ISC, theoretically, it should be possible to enhance the photophysical properties (Φ_T_, Φ_Δ_, and *τ*_T_) of any photosensitiser *via* metallation. In practice, this is not the case. Only one metallo-porphyrin photosensitiser (copper octaethylbenzochlorin) has shown photodynamic promise, the remaining efficient porphyrin photosensitisers are metal-free [13]. The reverse of this behaviour is observed for PCs and texaphyrins; only the (diamagnetic) metallated complexes have exhibited potential as photosensitisers [10, 12]. The metal-free analogues have shown no promise as photosensitisers [12].

**Expanded metallo-porphyrins**

Expanded porphyrins have a larger central binding cavity, increasing the number of potential metals it can accommodate.

Diamagnetic metallo-texaphyrins have shown encouraging photophysical properties; high triplet quantum yields and efficient generation of singlet oxygen [12, 64, 77]. In particular, the zinc and cadmium derivatives have shown triplet quantum yields close to unity [12]. In contrast, the paramagnetic metallo-texaphyrins, Mn-Tex, Sm-Tex, and Eu-Tex, have undetectable triplet quantum yields. This behaviour is parallel with that observed for the corresponding metallo-porphyrins [12].

The cadmium-texaphyrin derivative has shown *in vitro * photodynamic activity against human leukemia cells and Gram positive (*Staphylococcus*) and Gram negative (*Escherichia coli*) bacteria [98–101]. Although follow-up studies have been limited with this photosensitiser due to the toxicity of the complexed cadmium ion.

**Figure 27:**
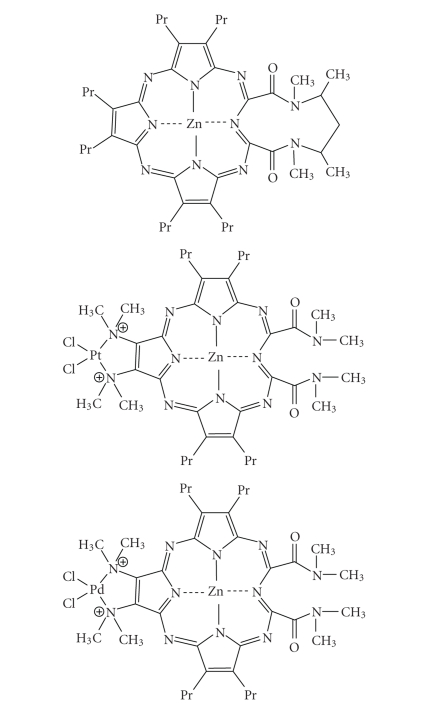
Zinc metallated *seco-*porphyrazine, platinum, and palladium derivatives.

A zinc-metallated *seco*-porphyrazine (Figure 27) has been developed with a high quantum singlet oxygen yield (Φ_Δ_ 0.74) [102]. This expanded porphyrin-like photosensitiser has shown the best singlet oxygen photosensitising ability of any of the reported *seco-*porphyrazines. Platinum and palladium derivatives have also been synthesised with singlet oxygen quantum yields of 0.59 and 0.54, respectively, (Figure 27) [102].

**Metallochlorins/bacteriochlorins**

The tin (IV) purpurins were found to be more active when compared with analogous zinc (II) purpurins, when evaluated against human cancers [5–7, 9, 18, 103, 104].

**Figure 28:**
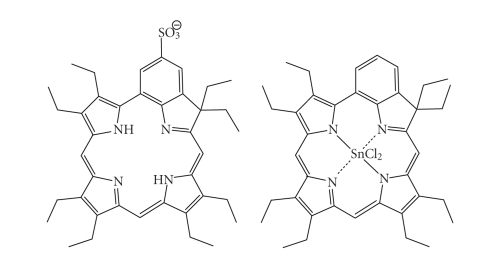
Sulphonated and tin (IV) benzochlorin derivatives.

Sulphonated benzochlorin derivatives have demonstrated a reduced phototherapeutic response against murine leukemia L1210 cells *in vitro* and transplanted urothelial cell carcinoma in rats, whereas the tin (IV) metallated benzochlorins exhibited an increased photodynamic effect in the same tumour model (Figure 28) [105].

The previously mentioned copper octaethylbenzochlorin (Figure 10) demonstrated an unexpected result. Despite an undetectable triplet state, it appears to be more *photoactive* towards leukemia cells *in vitro* and a rat bladder tumour model [106–108]. Suggestions for this unusual effect have pointed to interactions between the cationic iminium group and biomolecules [109]. Such interactions may allow electron-transfer reactions to take place *via* the short-lived excited singlet state and lead to the formation of radicals and radical ions. The copper-free derivative exhibited a tumour response with short intervals between drug administration and photodynamic therapy. Increased *in vivo* activity was observed with the zinc benzochlorin analogue [109].

**Metallo-phthalocyanines**

The photophysical properties of PCs are strongly influenced by the presence and nature of the central metal ion [12, 18, 64, 77]. Co-ordination of transition metal ions gives metallo-complexes with short triplet lifetimes (nanosecond range), resulting in different triplet quantum yields and lifetimes (with respect to the non-metallated analogues) [12]. The diamagnetic metals, such as zinc, aluminium, and gallium, generate metallo-phthalocyanines (MPC) with high triplet quantum yields (Φ_T_ ≥ 0.4) and short lifetimes (ZnPCS_4_ *τ*_T_ = 490 Fs and AlPcS_4_ *τ*_T_ = 400 Fs) and high singlet oxygen quantum yields (Φ_Δ_ ≥ 0.7) [12, 18, 64, 77, 110]. As a result, ZnPc and AlPc have been evaluated as second generation photosensitisers active against certain tumours [12].

**Metallo-naphthocyaninesulfobenzo-porphyrazines (M-NSBP)**

**Figure 29:**
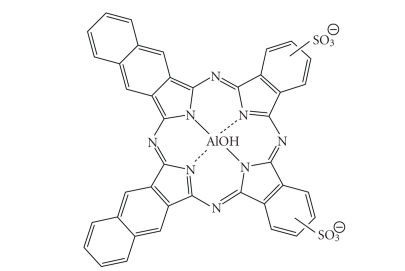
An aluminium naphthalocyaninesulfobenzoporphyrazine.

Aluminium has been successfully coordinated to M-NSBP (Figure 29). The resulting complex has shown photodynamic activity against EMT-6 tumour-bearing Balb/c mice (disulphonated analogue demonstrated greater photoactivity than the mono-derivative) [111].

**Metallo-naphthalocyanines**

**Figure 30:**
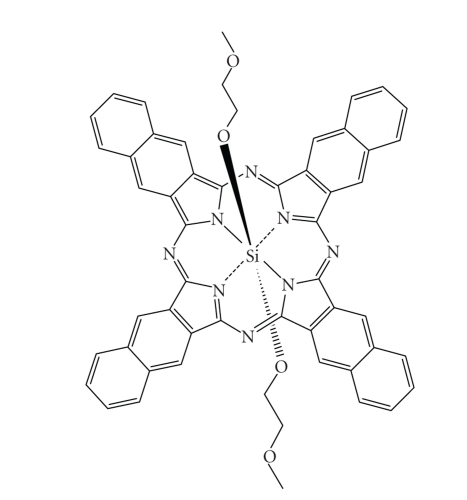
A siloxane naphthalocyanine.

Wöhrle and co-workers (Bulgaria) have concentrated their investigations on a zinc NC with various amido substituents. They observed the best phototherapeutic response (Lewis lung carcinoma in mice) with a tetrabenzamido analogue [112–114]. Kenney's group in the USA have studied complexes of silicon (IV) NCs (Figure 30) with two axial ligands in anticipation the ligands would minimise aggregation [115]. In particular, they investigated the disubstituted analogues as potential photodynamic agents [116, 117]. Kenney's results suggested that a siloxane NC substituted with two methoxyethyleneglycol ligands is an efficient photosensitiser against Lewis lung carcinoma in mice and that SiNC[OSi(i-Bu)_2_-n-C_18_H_37_]_2_ is effective against Balb/c mice MS-2 fibrosarcoma cells [118, 119]. van Lier and his group in Canada have also extensively investigated siloxane NCs as agents for photodynamic therapy [5, 76]. van Lier's research on these compounds suggests that they are efficacious photosensitisers against EMT-6 tumours in Balb/c mice also [120, 121]. The ability of certain metallo-NC derivatives (AlNc) to generate singlet oxygen is weaker than the analogous (sulphonated) metallo-PCs (AlPC); reportedly 1.6–3 orders of magnitude less [12].

It can be seen from the above examples that generalisation(s) between the nature of the parent chromophore; the presence/absence of a central metal ion; and the desirable photophysical properties required for a successful photosensitiser are difficult to make. In the porphyrin systems, the zinc ion appears to hinder the photodynamic activity of the compound; whereas, in the higher/expanded *π*-systems, dyes chelated with the same metal ion are observed to form complexes with good to high photophysical/photodynamic properties.

In order to try and address these observations, Sessler and his group undertook an extensive study into the metallated texaphyrins, investigating the “influence of large metal cations on the photophysical properties of texaphyrins.” They particularly studied “the effect of metal cations on the photophysical properties of coordinating ligands.” The group concentrated on the lanthanide (III) metal ions, Y, In, Lu, Cd, Nd, Sm, Eu, Gd, Tb, Dy, Ho, Er, Tm, and Yb [97].

Sessler and co-workers observed that when diamagnetic Lu (III) was complexed to texaphyrin, an effective photosensitiser (Lutex) was generated. When they substituted the paramagnetic Gd (III) ion for the Lu metal, photodynamic activity was lost. As a result, the group investigated a range of diamagnetic and paramagnetic ions [97].

Sessler further reported a correlation between the excited-singlet and triplet state lifetimes and the rate of ISC of the diamagnetic texaphyrin complexes, Y(III), In (III), and Lu (III), and the atomic number of the cation [97].

Paramagnetic metallo-texaphyrins were observed to display rapid ISC. Greater effects on the rates of triplet decay were also observed, and the triplet lifetimes were strongly affected by the choice of metal centre [97]. The diamagnetic ions (Y, In, and Lu) were recorded as having triplet lifetimes ranging from 187, 126, and 35 *μ*s, respectively. Comparable lifetimes for the paramagnetic species (Eu-Tex 6.98 *μ*s, Gd-Tex 1.11, Tb-Tex < 0.2, Dy-Tex 0.44 × 10^−3^, Ho-Tex 0.85 × 10^−3^, Er-Tex 0.76 × 10^−3^, Tm-Tex 0.12 × 10^−3^, and Yb-Tex 0.46) were obtained [97].

Sessler and his group were only able to measure the triplet quantum yields for three of the paramagnetic complexes (see Table 1). The results were significantly lower than the diamagnetic metallo-texaphyrins [97].

The data collected from Sessler and co-workers experiments suggests that, in general, singlet oxygen quantum yields closely follow the triplet quantum yields.

Their experimental data leads to the conclusion that various diamagnetic and paramagnetic texaphyrins investigated have independent photophysical behaviour with respect to a complex's magnetism. The diamagnetic complexes were characterised by relatively high fluorescence quantum yields, excited-singlet and triplet lifetimes, and singlet oxygen quantum yields; in distinct contrast to the paramagnetic species investigated [97].

Results suggested that the +2 charged diamagnetic species exhibit a direct relationship between their fluorescence quantum yields, excited state lifetimes, rate of ISC, and the atomic number of the metal ion. The greatest diamagnetic ISC rate was observed for Lu-Tex; a result ascribed to the heavy atom effect. The heavy atom effect also held for the Y-Tex, In-Tex, and Lu-Tex triplet quantum yields and lifetimes. The triplet quantum yields and lifetimes both decreased with increasing atomic number. The singlet oxygen quantum yield correlated with this observation [97].

The photophysical properties displayed by the paramagnetic species were more complex. A simple correlation between the observed data/behaviour and the number of unpaired electrons located on the metal ion could not be made. For example, the ISC rates and the fluorescence lifetimes gradually decreased with increasing atomic number, the Gd-Tex, and Tb-Tex chromophores showed (despite having a larger number of unpaired electrons) slower rates of ISC and longer lifetimes than Ho-Tex or Dy-Tex. Sessler suggested that charge transfer or intermolecular energy transfer is taking place from higher excited states (such as S_2_) [97].

## 12. SUMMARY

A variety of second generation photosensitisers have been developed and evaluated against a range of clinical applications (see Tables 2 and 3). The metallation of a number of these chromophores has generated a variety of photosensitisers with improved photophysical properties. The effectiveness of these metallo-photosensitisers depends largely (but not definitively) on the nature of the co-ordinated central metal ion. Chromophores chelated to diamagnetic transition metals and lanthanide ions have shown the greatest potential as photodynamic agents, a consequence of the heavy metal effect enhancing the rate of ISC. As a result, a number of these metallated tetrapyrrole-based macrocycles are currently photosensitisers of choice, particularly the zinc (II), aluminium (III), and tin (IV) complexes.

## Figures and Tables

**Box 1:**
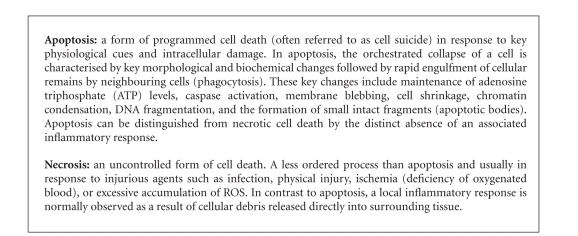


**Box 2:**
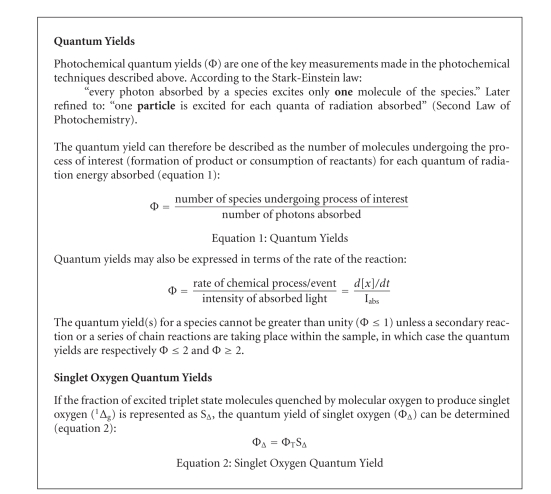


**Box 3:**
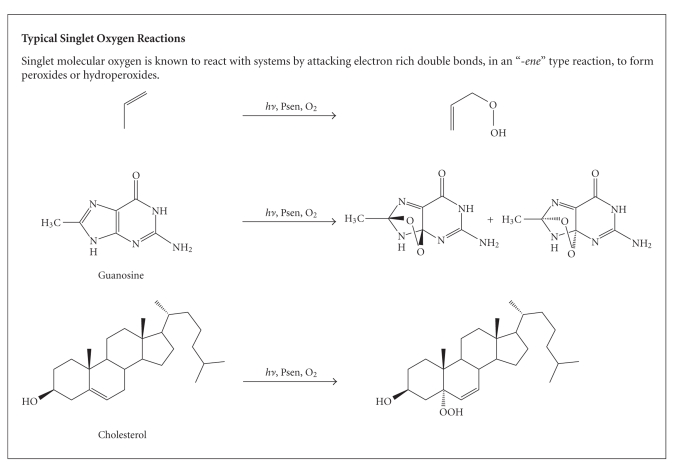


**Box 4:**
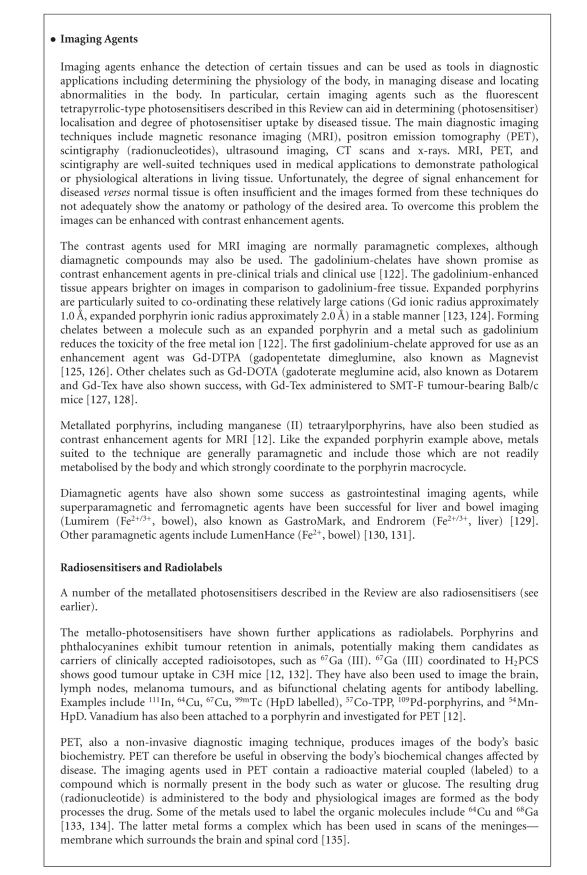


**Table 1:** Quantum triplet yields of texaphyrin metallated with paramagnetic and diamagnetic lanthanides (data reproduced from [97]).

Paramagnetic MTex	Φ_T_		Diamagnetic MTex
Eu-Tex	0.090	0.563	Y-Tex
Gd-Tex	0.156	0.500	In-Tex
Yb-Tex	0.126	0.340	Lu-Tex

**Table 2:** Summary of a collection of different photosensitiser types and their absorption data.

CLASS OF PHOTOSENSITISER	LONGEST WAVELENGTH ABSORPTION/nm	EXTINCTION COEFFICIENT/ M^−1^cm^−1^	DRUGS IN CLINICAL TRIAL (Phase I–III)	DRUGS APPROVED FOR PDT (PRECLINICAL AND CLINICAL)
Porphyrins	620–640	3,500	—	Photofrin
				Levulan
				Metvix

(Expanded Porphyrins)				
Porphycenes	610–650	50,000		ATMPn
Texaphyrins	730–770	40,000	Lu-Tex	
			Optrin	
			Antrin	
			Xcytrin	
			Benzvix	
			Hexvix	

Chlorins	650–690	40,000	Foscan	Visudyne
			Puryltin	

Bacteriochlorins	730–800	150,000	—	—

Phthalocyanines	680–780	200,000	CGP55847	—
			PC4	
			Photosense	

Naphthalocyanines	740–780	250,000	—	—

**Table 3:** Summary of a range of photosensitisers and their clinical applications.

TRADE NAME	MARKETING COMPANY	PRE-/CLINICAL APPLICATION	COUNTRIES APPROVED IN
Photofrin	QLT Phototherapeutics	Oesophageal, lung, bladder and cervical dysplasia	Canada (1993),
			The Netherlands (1994),
			Japan (1994),
			USA (1995),
			France (1996),
			Germany (1997),
			Finland (1999),
			UK (1999),
			Sweden (2000),
			Italy (2000),
			Ireland (2000),
			Poland (2000)

Levulan	DUSA Pharmaceuticals	Actinic keratosis	USA (1999),
		Actinic keratosis and basal-cell carcinoma	Sweden (2001),
			Europe (2001)

Metvix	Photocure ASA	Actinic keratosis and basal-cellcarcinoma	Sweden (2001),
			Europe (2001)

Visudyne	QLT Phototherapeutics	Wet-AMD	Europe (2001),
			USA (2000),
			Canada (2000),
		Subfoveal choroidal neovascularisation	Europe (2001),
			USA(2001),
			Canada (2001)

ATMPn	GlaxoWellcome and Cytopharm	Psorasis and non-melanoma skin cancer	Germany (1997)

Purlytin	Miravant Medical Technologies	Psorasis and restenosis	USA (1998)

Foscan	BioLitec Pharmaceuticals	Head and neck cancers	Europe (2001)

## GLOSSARY

1. AMD is the leading cause of blindness in humans over the age of 50. AMD is characterised by a rapid growth of abnormal blood vessels under the central retina causing scarring, and an accelerated loss of visual acuity [58].
2. Chemosensitisers are drugs or chemicals which are used to enhance the therapeutic effects of anti-cancer (chemotherapy) drugs. They make the tumour cells more sensitive to the effects of chemotherapy.
3. Diamagnetic species is a species with no unpaired electrons, that is, all electrons are spin-paired.
4. Fluorophore is generally a molecule capable of absorbing light energy when irradiated at a specific wavelength and emitting energy at longer wavelengths.
5. Heavy atom effect enhances coupling between the excited-singlet (S_1_) and excited-triplet (T_1_) states. It is the enhancement of a spin-forbidden process by the presence of an atom of high atomic molecular weight. Mechanistically, it responds to a spin-orbit coupling enhancement produced by a heavy atom. Spin-forbidden and spin-allowed processes are highlighted in Section 7.
6. Luminescence lifetime is the average time a molecule spends in an excited state (S_*n*_ > 0 or T_*n*_ > 0).
7. Methylene blue is used to sterilize/decontaminate freshly frozen plasma units by inactivating extracellularly enveloped viruses (such as HIV), as well as methaemoglobinaemia [5, 9].
8. Paramagnetic species is a species with one or more unpaired electrons.
9. Phase I clinical trials are used to determine pharmacokinetic properties (metabolism, elimination, and preferred method of administration) and a safe dosage range, and identify any side effects of a new drug. They are performed on a small number of people (20–80).
10. Phase II clinical trials are performed on a larger group of people (100–300). The drug is further evaluated to test its effectiveness and any side effects.
11. Phase III clinical trials: the drugs effectiveness is confirmed and comparisons are made to more commonly used treatment modalities in a range of 1000–3000 people. Potential side effects are monitored.
12. Phase IV clinical trials are post-marketing observations and evaluations.
13. Photochemotherapy is a combination of a chemical substance and light to treat disease.
14. Phototherapy is a term used to describe the treatment of disease by a series of (photo-) chemical processes initiated by light.
15. Radiosensitisers are drugs which boost the effect of radiation therapy (radiotherapy) by making the tumour tissue more vulnerable to the applied radiation. They may be used alone or in conjunction with other drugs.
16. Restenosis is the renarrowing of a coronary artery after angioplasty or stenting.

## References

[1] Edelson MF (1988). Light-activated drugs. *Scientific American*.

[2] Sternberg ED, Dolphin D, Brückner C (1998). Porphyrin-based photosensitizers for use in photodynamic therapy. *Tetrahedron*.

[3] Bonnett R, Martinez G (2001). Photobleaching of sensitisers used in photodynamic therapy. *Tetrahedron*.

[4] Allison RR, Mota HC, Sibata CH (2004). Clinical PD/PDT in North America: an historical review. *Photodiagnosis and Photodynamic Therapy*.

[5] Sharman WM, Allen CM, van Lier JE (1999). Photodynamic therapeutics: basic principles and clinical applications. *Drug Discovery Today*.

[6] MacDonald IJ, Dougherty TJ (2001). Basic principles of photodynamic therapy. *Journal of Porphyrins and Phthalocyanines*.

[7] Dougherty TJ, Gomer CJ, Henderson BW (1998). Photodynamic therapy. *Journal of the National Cancer Institute*.

[8] Bonnett R (1995). Photosensitizers of the porphyrin and phthalocyanine series for photodynamic therapy. *Chemical Society Reviews*.

[9] Detty MR, Gibson SL, Wagner SJ (2004). Current clinical and preclinical photosensitizers for use in photodynamic therapy. *Journal of Medicinal Chemistry*.

[10] Kaestner L, Cesson M, Kassab K (2003). Zinc octa-n-alkyl phthalocyanines in photodynamic therapy: photophysical properties, accumulation and apoptosis in cell cultures, studies in erythrocytes and topical application to Balb/c mice skin. *Photochemical & Photobiological Sciences*.

[11] Brown SB, Brown EA, Walker I (2004). The present and future role of photodynamic therapy in cancer treatment. *Lancet Oncology*.

[12] Ali H, van Lier JE (1999). Metal complexes as photo- and radio-sensitizers. *Chemical Reviews*.

[13] Selman SH, Hampton JA, Morgan AR (1993). Copper benzochlorin, a novel photosensitizer for photodynamic therapy: effects on a transplantable urothelial tumor. *Photochemistry and Photobiology*.

[14] Mir Y, Houde D, van Lier JE (2006). Two-photon absorption of copper tetrasulfophthalocyanine induces phototoxicity towards Jurkat cells *in vitro*. *Photochemical & Photobiological Sciences*.

[15] Villanueva A, Jori G (1993). Pharmacokinetic and tumour-photosensitizing properties of the cationic porphyrin *meso*-tetra(4N-methylpyridyl)porphine. *Cancer Letters*.

[16] Sessler JL, Tvermoes NA, Davis J (1999). Expanded porphyrins. Synthetic materials with potential medical utility. *Pure and Applied Chemistry*.

[17] Castano AP, Demidova TN, Hamblin MR (2004). Mechanisms in photodynamic therapy: part one—photosensitizers, photochemistry, and cellular localization. *Photodiagnosis and Photodynamic Therapy*.

[18] Nyman ES, Hynninen PH (2004). Research advances in the use of tetrapyrrolic photosensitizers for photodynamic therapy. *Journal of Photochemistry and Photobiology B*.

[19] Svanberg K, Wang I, Colleen S (1998). Clinical multi-colour fluorescence imaging of malignant tumours-initial experience. *Acta Radiologica*.

[20] Smetana Z, Ben-Hur E, Mendelson E, Salzberg S, Wagner P, Malik Z (1998). Herpes simplex virus proteins are damaged following photodynamic inactivation with phthalocyanines. *Journal of Photochemistry and Photobiology B*.

[21] Jori G (1996). Tumour photosensitizers: approaches to enhance the selectivity and efficiency of photodynamic therapy. *Journal of Photochemistry and Photobiology B*.

[22] Decreau R, Richard MJ, Verrando P, Chanon M, Julliard M (1999). Photodynamic activities of silicon phthalocyanines against achromic M6 melanoma cells and healthy human melanocytes and keratinocytes. *Journal of Photochemistry and Photobiology B*.

[23] Hudson R, Carcenac M, Smith K (2005). The development and characterisation of porphyrin isothiocyanate-monoclonal antibody conjugates for photoimmunotherapy. *British Journal of Cancer*.

[24] Malatesti N, Smith K, Savoie H, Greenman J, Boyle RW (2006). Synthesis and *in vitro* investigation of cationic 5,15-diphenyl porphyrin-monoclonal antibody conjugates as targeted photodynamic sensitisers. *International Journal of Oncology*.

[25] Staneloudi C, Smith KA, Hudson R (2007). Development and characterization of novel photosensitizer: scFv conjugates for use in photodynamic therapy of cancer. *Immunology*.

[26] Rebeiz CA, Reddy KN, Nandihalli UB, Velu J (1990). Tetrapyrrole-dependent photodynamic herbicides. *Photochemical and Photobiological*.

[27] BenAmor T, Tronchin M, Bortolotto L, Verdiglione R, Jori G (1998). Porphyrins and related compounds as photoactivatable insecticides – phototoxic activity of hematoporphyrin towards Ceratitis capitata and Bactrocera oleae. *Photochemistry and Photobiology*.

[28] Sessler JL, Cyr MJ, Maiya BG Photodynamic inactivation of enveloped viruses using sapphyrin, a 22 pi-electron expanded porphyrin: possible approaches to prophylactic blood purification protocols.

[29] Keating PB, Hinds MF, Davis SJ A singlet oxygen sensor for photodynamic cancer therapy.

[30] Wayne CE, Wayne RP (1996). Photochemical principles. *Photochemistry*.

[31] Wayne CE, Wayne RP (1996). Photophysics. *Photochemistry*.

[32] Schweitzer C, Schmidt R (2003). Physical mechanisms of generation and deactivation of singlet oxygen. *Chemical Reviews*.

[33] Gilbert A, Baggott J (1991). *Essentials of Molecular Photochemistry*.

[34] Gilbert A, Baggott J (1991). *Essentials of Molecular Photochemistry*.

[35] Kochevar IE, Redmond RW (2000). Photosensitized production of singlet oxygen. *Methods in Enzymology*.

[36] Lang K, Mosinger J, Wagnerová DM (2004). Photophysical properties of porphyrinoid sensitizers non-covalently bound to host molecules; models for photodynamic therapy. *Coordination Chemistry Reviews*.

[37] van Lier JE, Valenzeno DP, Pottier RH, Mathis P, Douglas RH (1991). Photosensitization: reaction pathways. *Photobiological Techniques, Photosensitisation: Reaction Pathways*.

[38] Wilkinson F, Helman WP, Ross AB (1993). Quantum yields for the photosensitized formation of the lowest
electronically excited singlet state of molecular oxygen in
solution. *Journal of Physical and Chemical Reference Data*.

[39] Cló E,  Snyder JW, Ogilby PR, Gothelf KV (2007). Control and selectivity of photosensitized singlet oxygen production: challenges in complex 
biological systems. *ChemBioChem*.

[40] Snyder JW, Skovsen E, Lambert JDC, Poulsen L, Ogilby PR (2006). Optical detection of singlet oxygen from single cells. *Physical Chemistry Chemical Physics*.

[41] Skovsen E, Snyder JW, Lambert JDC, Ogilby PR (2005). Lifetime and diffusion of singlet oxygen in a cell. *Journal of Physical Chemistry B*.

[42] Castano AP, Demidova TN, Hamblin MR (2005). Mechanisms in photodynamic therapy: part two—cellular signaling, cell metabolism and modes of cell death. *Photodiagnosis and Photodynamic Therapy*.

[43] Oschner M (1997). Photophysical and photobiological processes in the photodynamic therapy of tumours. *Journal of Photochemistry and Photobiology B*.

[44] Weizman E, Rothmann C, Greenbaum L (2000). Mitochondrial localization and photodamage during photodynamic therapy with tetraphenylporphines. *Journal of Photochemistry and Photobiology B*.

[45] www.chem.ucla.edu/dept/Organic/CSF_Brochure.html.

[46] See KL, Forbes IJ, Betts WH (1984). Oxygen dependency of photocytotoxicity with haematoporphyrin derivative. *Photochemistry and Photobiology*.

[47] Ma LW, Moan J, Berg K (1994). Evaluation of a new photosensitizer, *meso*-tetra-hydroxyphenyl-chlorin, for use in photodynamic therapy. A comparison of its photobiological properties with those of two other photosensitizers. *International Journal of Cancer*.

[48] Hasan T, Khan AU (1986). Phototoxicity of the tetracyclines: photosensitized emission of singlet delta dioxygen. *Proceedings of the National Academy of Sciences of the United States of America*.

[49] Morgan J, Oseroff AR (2001). Mitochondria-based photodynamic anti-cancer therapy. *Advanced Drug Delivery Reviews*.

[50] Anderson HL (1999). Building molecular wires from the colours of life: conjugated porphyrin oligomers. *Chemical Communications*.

[51] Milgrom LR (1997). What porphyrins are and what they do. *The Colours of Life: An Introduction to the Chemistry of Porphyrins and Related Compounds*.

[52] Milgrom LR (1997). How do they do it? —making oxygen. *The Colours of Life: An Introduction to the Chemistry of Porphyrins and Related Compounds*.

[53] Rest AJ (1982). Porphyrins and phthalocyanines. *Light, Chemical Changes and Life*.

[54] Rimmington C (1960). Spectral absorption coefficients of some porphyrins in the Soret-band region. *Journal of Biochemistry*.

[55] http://chemgroups.ucdavis.edu/~smith/chime/Porph_Struct/lots_of_files/intro.html.

[56] Bonnett R, Charlesworth P, Djelal BD, Foley S, McGarvey DJ, Truscott TG (1999). Photophysical properties of 5,10,15,20-tetrakis(*m*-hydroxyphenyl)porphyrin (m-THPP), 5,10,15,20-tetrakis(*m*-hydroxyphenyl)chlorin (*m*-THPC) and 5,10,15,20-tetrakis(*m*-hydroxyphenyl)bacteriochlorin (m-THPBC): a comparative study. *Journal of the Chemical Society. Perkin Transactions II*.

[57] Richter AM, Kelly B, Chow J (1987). Preliminary studies on a more effective phototoxic agent than hematoporphyrin. *Journal of the National Cancer Institute*.

[58] van den Bergh H, Sickenberg M, Ballini J-P (1998). *International Photodynamic Therapy*.

[59] Levy JG, Jones CA, Pilson LA (1994). The preclinical and clinical development and potential application of benzoporphyrin derivative. *International Photodynamic Therapy*.

[60] Aveline BM, Hasan T, Redmond RW (1995). The effects of aggregation, protein binding and cellular incorporation on the photophysical properties of benzoporphyrin derivative monoacid ring A (BPDMA). *Journal of Photochemistry and Photobiology B*.

[61] Stables GI, Ash DV (1995). Photodynamic therapy. *Cancer Treatment Reviews*.

[62] Morgan AR, Garbo GM, Keck RW, Selman SH (1988). New photosensitizers for photodynamic therapy: combined effect of
metallopurpurin derivatives and light on transplantable bladder tumors. *Cancer Research*.

[63] Kübler A, Haase T, Staff C, Kahle B, Rheinwald M, Mühling J (1999). Photodynamic therapy of primary nonmelanomatous skin tumours of the head and neck. *Lasers in Surgery and Medicine*.

[64] Ball DJ, Wood SR, Vernon DI, Griffiths J, Dubbelman TMAR, Brown SB (1998). The characterisation of three substituted zinc phthalocyanines of differing charge for use in photodynamic therapy. a comparative study of their aggregation and photosensitising ability in relation to *m*
-THPC and polyhaematoporphyrin. *Journal of Photochemistry and Photobiology B*.

[65] Sessler JL, Johnson MR, Lynch V (1987). Synthesis and crystal structure of a novel tripyrrane-containing porphyrinogen-like macrocycle. *Journal of Organic Chemistry*.

[66] Sessler JL, Hemmi G, Mody TD, Murai T, Burrell A, Young SW (1994). Texaphyrins: synthesis and applications. *Accounts of Chemical Research*.

[67] Woodburn KW, Fan Q, Kessel D, Luo Y, Young SW (1998). Photodynamic therapy of B16F10 murine melanoma with lutetium texaphyrin. *The Journal of Investigative Dermatology*.

[68] Woodburn KW, Qing F, Kessel D, Young SW Photoeradication and imaging of atheromatous plaque with texaphyrins.

[69] Szeimies R-M, Karrer S, Abels C (1996). 9-Acetoxy-2,7,12,17-tetrakis(*β*-methoxyethyl)-porphycene (ATMPn), a novel photosensitizer for photodynamic therapy: uptake kinetics and intracellular localization. *Journal of Photochemistry and Photobiology B*.

[70] Kimmel S, Gottfried V, Davidi R, Averbuj C *In vivo* uptake and photodynamic activity of porphycenes.

[71] Aicher A, Miller K, Reich ED, Hautmann RE (1993). Photosensitization of human bladder carcinoma cells *in vitro* by 9-acetoxy-tetra-n-proylporphycence (ATPPn) bound to liposomes from soya phosphatidylcholine. *Optical Engineering*.

[72] Karrer S, Abels C, Szeimies R-M (1997). Topical application of a first porphycene dye for photodynamic therapy—penetration studies in human perilesional skin and basal cell carcinoma. *Archives of Dermatological Research*.

[73] Oschner M (1996). Light scattering of human skin: a comparison between zinc(II)-phthalocyanine and photofrin II^®^. *Journal of Photochemistry and Photobiology B*.

[74] Schieweck K, Capraro H-G, Isele U CGP 55 847, liposome-delivered zinc(II)-phthalocyanine as a phototherapeutic agent for tumors.

[75] Cook MJ (2002). Properties of some alkyl substituted phthalocyanines and related macrocycles. *The Chemical Record*.

[76] Fabris C, Soncin M, Miotto G (2006). Zn(II)-phthalocyanines as phototherapeutic agents for cutaneous diseases. Photosensitization of fibroblasts and keratinocytes. *Journal of Photochemistry and Photobiology B*.

[77] Mantareva V, Kussovski V, Angelov I (2007). Photodynamic activity of water-soluble phthalocyanine zinc(II) complexes against pathogenic microorganisms. *Bioorganic & Medicinal Chemistry*.

[78] Cauchon N, Nader M, Bkaily G, van Lier JE, Hunting D (2006). Photodynamic activity of substituted zinc trisulfophthalocyanines: role of plasma membrane damage. *Photochemistry and Photobiology*.

[79] Sobolev AS, Stranadko EF (1997). *International Photodynamic Therapy*.

[80] Sokolov VV, Chissov VI, Filonenko EV First clinical results with a new drug for PDT.

[81] Zharkova NN, Kozlov DN, Smirnov VV Fluorescence observations of patients in the course of photodynamic therapy of cancer with the photosensitizer PHOTOSENS.

[82] Phillips D (1995). The photochemistry of sensitizers for photodynamic therapy. *Pure and Applied Chemistry*.

[83] Oleinick NL, Antunez AR, Clay ME, Rihter BD, Kenney ME (1993). New phthalocyanine photosensitizers for photodynamic therapy. *Photochemistry and Photobiology*.

[84] Whitacre CM, Feyes DK, Satoh T (2000). Photodynamic therapy with the phthalocyanine photosensitizer PC4 of SW480
human colon cancer xenografts in athymic mice. *Clinical Cancer Research*.

[85] Lo P-C, Leung SCH, Chan EYM, Fong W-P, Ko W-H, Ng DNP (2007). Photodynamic effects of a novel series of silicon(IV) phthalocyanines against human colon adenocarcinoma cells. *Photodiagnosis and Photodynamic Therapy*.

[86] Zaidi SIA, Agarwal R, Eichler G, Rihter BD, Kenney ME, Mukhtar H (1993). Photodynamic effects of new silicon phthalocyanines – *in vitro* studies utilising rat hepatic microsomes and human erythrocyte-ghosts as model membrane sources. *Journal of Photochemistry and Photobiology B*.

[87] Colussi VC, Feyes DK, Mulvihill JW (1999). Phthalocyanine 4 (Pc4) photodynamic therapy of human OVCAR-3 tumour xenografts. *Photochemistry and Photobiology*.

[88] Whitacre CM, Satoh TH, Xue L-Y, Gordon NH, Oleinick NL (2002). Photodynamic therapy of human breast cancer xenografts lacking caspase-3. *Cancer Letters*.

[89] George JE, Ahmad Y, Varghai D (2005). PC4 photodynamic therapy of U87-derived human glioma in the nude rat. *Lasers in Surgery and Medicine*.

[90] Kaplan ML, Lovinger AJ, Reents WD, Schmidt PH (1984). The preparation, spectral properties, and x-ray structural features of 2,3-naphthalocyanines. *Molecular Crystals and Liquid Crystals*.

[91] Brunner H, Obermeier H, Szeimies R-M (1995). Platin(II)-komplexe mit porphyrinliganden: synthese und synergismen bei der photodynamischen tumor therapie. *Chemische Berichte*.

[92] Ding L, Casas C, Etemad-Moghadam G, Meunier B, Cros S (1990). Synthesis of water-soluble, cationic functionalised metalloporphyrins having a cytotoxic activity. *New Journal of Chemistry*.

[93] Ding L, Balzarini J, Schols D, Meunier B, De Clercq EB (1992). Anti-human immunodeficiency virus effects of cationic metalloporphyrin-ellipticine complexes. *Biochemical Pharmacology*.

[94] Wöhrle D, Hirth A, Bogdahn-Rai T, Schnurpfeil G, Shopova M (1998). Photodynamic therapy of cancer: second and third generations of photosensitizers. *Russian Chemical Bulletin*.

[95] Alvarez MG, Vittar NBR, Principe F (2004). Pharmacokinetic and phototherapeutic studies of monocationic methoxyphenylporphyrin derivative. *Photodiagnosis and Photodynamic Therapy*.

[96] Collins-Gold L, Feichtinger N, Wärnheim T (2000). Are lipid emulsions the drug delivery solution?. *Modern Drug Discovery*.

[97] Guldi DM, Mody TD, Gerasimchuk NN, Magda D, Sessler JL (2000). Influence of large metal cations on the photophysical properties of texaphyrin, a rigid aromatic chromophore. *Journal of the American Chemical Society*.

[98] Harriman A, Maiya BG, Murai T, Hemmi G, Sessler JL, Mallouk TE (1989). Metallotexaphyrins: a new family of photosensitisers for efficient generation of singlet oxygen. *Journal of the Chemical Society. Chemical Communications*.

[99] Sessler JL, Hemmi G, Maiya BG Tripyrroledimethine-derived (texaphyrin-type) macrocycles: potential photosensitizers which absorb in the far-red spectral region.

[100] Ehrenberg B, Malik Z, Nitzan Y (1993). The binding and photosensitization effects of tetrabenzoporphyrins and texaphyrin in bacterial cells. *Lasers in Medical Science*.

[101] Ehrenberg B, Roitman L, Lavi A, Nitzan Y, Malik Z, Sessler JL Spectroscopic studies of photosensitization in solutions and in cells.

[102] Garrido-Montalban A, Baum SM, Barrett AGM, Hoffman BM (2003). Studies on *seco*-porphyrazines: a case study on serendipity. *Dalton Transactions*.

[103] Garbo GM (1996). Purpurins and benzochlorins as sensitizers for photodynamic therapy. *Journal of Photochemistry and Photobiology B*.

[104] Razum NJ, Snyder AB, Doiron DR SnET2: clinical update.

[105] Kessel D, Morgan AR (1993). Photosensitization with etiobenzochlorins and octaethylbenzochlorins. *Photochemistry and Photobiology*.

[106] Selman SH, Hampton JA, Morgan AR, Keck RW, Balkany AD, Skalkos D (1993). Copper benzochlorin, a novel photosensitiser for photodynamic therapy – effects on a transplantable urothelial tumour. *Photochemistry and Photobiology*.

[107] Hampton JA, Skalkos D, Taylor PM, Selman SH (1993). Iminium salt of copper benzochlorin (CDS1), a novel photosensitizer for photodynamic therapy: mechanism of cell killing. *Photochemistry and Photobiology*.

[108] Skalkos D, Hampton JA, Keck RW, Wagoner M, Selman SH (1994). Iminium salt benzochlorins: structure-activity relationship studies. *Photochemistry and Photobiology*.

[109] Garbo GM, Fingar VH, Weiman TJ (1998). *In vivo* and *in vitro* photodynamic studies with benzochlorin iminium salts delivered by a lipid emulsion. *Photochemistry and Photobiology*.

[110] Reddi E, LoCastro G, Biolo R, Jori G (1987). Pharmacokinetic studies with zinc(II)-phthalocyanine in tumour-bearing mice. *British Journal of Cancer*.

[111] Margaron P, Langlois R, van Lier JE, Gaspard SJ (1992). Photodynamic properties of naphthosulfobenzoporphyrazines, novel asymmetric, amphiphilic phthalocyanine derivatives. *Photochemistry and Photobiology*.

[112] Wöhrle D, Shopova M, Müller S (1993). Liposome-delivered Zn(II)-2,3-naphthalocyanines as potential sensitizers for PDT: synthesis, photochemical, pharmacokinetic, and phototherapeutic studies. *Journal of Photochemistry and Photobiology B*.

[113] Shopova M, Wöhrle D, Stoichkova N (1994). Hydrophobic Zn(II)-naphthalocyanines as photodynamic therapy agents for Lewis-lung carcinoma. *Journal of Photochemistry and Photobiology B, Biology*.

[114] Müller S, Mantareva VN, Stoichkova N (1996). Tetraamido-substituted 2,3-napthalocyanine zinc(II) complexes as phototherapeutic agents: Synthesis, comparative photochemical and photobiological studies. *Journal of Photochemistry and Photobiology B*.

[115] Wheeler BL, Nagasubramanian G, Bard AJ, Schechtman LA, Dininny DR, Kenney ME (1984). A silicon phthalocyanine and a silicon naphthalocyanine: synthesis, electrochemistry, and electrogenerated chemiluminescence. *Journal of the American Chemical Society*.

[116] Zuk MM, Rihter BD, Kenney ME, Rodgers MAJ, Kreimer-Birnbaum M (1994). Pharmacokinetic and tissue distribution studies of the photosensitizer bis(di-isobutyl octadecylsiloxy)silicon
2,3-naphthalocyanine (isobosinc) in normal and tumor-bearing rats. *Photochemistry and Photobiology*.

[117] Zuk MM, Rihter BD, Kenney ME, Rodgers MAJ, Kreimer-Birnbaum M (1996). Effect of delivery system on the pharmacokinetics and tissue
distribution of bis(di-isobutyl octadecylsiloxy)silicon
2,3-naphthalocyanine (isobosinc), a photosensitizer for tumor
therapy. *Photochemistry and Photobiology*.

[118] Cuomo V, Jori G, Rihter B, Kenney ME, Rodgers MAJ (1990). Liposome-delivered Si(IV)-naphthalocyanine as a photodynamic sensitiser for experimental tumours: pharmacokinetic and phototherapeutic studies. *British Journal of Cancer*.

[119] Mantareva VN, Shopova M, Spassova G (1997). Si(IV)-methoxyethylene-glycol-naphthalocyanine: synthesis and pharmacokinetic and photosensitizing properties in different tumour models. *Journal of Photochemistry and Photobiology B*.

[120] Brasseur N, Nguyen T-L, Langlois R (1994). Synthesis and photodynamic activities of silicon 2,3-naphthalocyanine derivatives. *Journal of Medicinal Chemistry*.

[121] Brasseur N, Ouellet R, Lewis K, Potter WR, van Lier JE (1995). Photodynamic activities and skin photosensitivity of the
bis(dimethylthexylsiloxy)silicon 2,3-naphthalocyanine in mice. *Photochemistry and Photobiology*.

[122] Raymond KN, Pierre VC (2005). Next generation, high relaxivity gadolinium MRI agents. *Bioconjugate Chemistry*.

[123] Sessler JL, Burrell AK (1992). Expanded porphyrins. *Topics in Current Chemistry*.

[124] Sheldon RA, Sheldon RA *Metalloporphyrins in Catalytic Oxidations*.

[125] Schmidt HC, McNamara MT, Brasch RC, Higgins CB (1985). Assessment of severity of experimental pulmonary edema with magnetic resonance imaging. Effect of relaxation enhancement by Gd-DTPA. *Investigative Radiology*.

[126] Curati WL, Graif M, Kingsley DP, Niendorf HP, Young IR (1986). Acoustic neuromas: Gd-DTPA enhancement in MR imaging. *Radiology*.

[127] Magerstädt M, Gansow OA, Brechbiel MW (1986). Gd(DOTA): an alternative to Gd(DTPA) as a T[1,2] relaxation agent for NMR imaging or spectroscopy. *Magnetic Resonance in Medicine*.

[128] Sessler JL, Mody TD, Hemmi GW, Lynch V, Young SW, Miller RA (1993). Gadolinium(III) texaphyrin: a novel MRI contrast agent. *Journal of the American Chemical Society*.

[129] Wang Y-XJ, Hussain SM, Krestin GP (2001). Superparamagnetic iron oxide contrast agents: physicochemical characteristics and applications in MR imaging. *European Radiology*.

[130] Small WC, DeSimone-Macchi D, Parker JR (1999). A mult-isite phase III study of the safety and efficacy of a new manganese chloride-based gastrointestinal contrast agent for MRI of the abdomen and pelvis. *Journal of Magnetic Resonance Imaging*.

[131] Schwert DD, Davies JA, Richardson N (2002). Non-gadolinium-based MRI contrast agents. *Topics in Current Chemistry*.

[132] Rousseau J, Boyle RW, MacLennan AH, Truscott TG, van Lier JE (1991). Biodistribution and tumor uptake of [Ga-67] chlorogallium-tetraoctadecyloxy phthalocyanine and its sulfonation products in tumor bearing C-3H mice. *Nuclear Medicine and Biology*.

[133] Cai W, Chen K, Mohamedali KA (2006). PET of vascular endothelial growth factor receptor expression. *The Journal of Nuclear Medicine*.

[134] Schuhmacher J, Zhang HW, Doll J (2005). GRP receptor-targeted PET of a rat pancreas carcinoma xenograft in nude mice with a ^68^Ga-labeled bombesin(6-14) analog. *Journal of Nuclear Medicine*.

[135] Henze M, Dimitrakopoulou-Strauss A, Milker-Zabel S (2005). Characterization of ^68^Ga-DOTA-D-Phe1-Tyr3-octreotide kinetics in patients with meningiomas. *Journal of Nuclear Medicine*.

